# Supplementary material for: Data in support of quantification of pyrophosphate as a universal approach to determine polymerase activity and assay polymerase inhibitors
Source: Data Brief. 2015 Apr 22;4:14–8. doi: 10.1016/j.dib.2015.04.006 (PMC4510374; doi:10.1016/j.dib.2015.04.006)
Supplement: Supplementary file 1 — Supplementary material [file mmc1.zip › Data in Brief .docx]

*Data article*

**Title:** Data in support of quantification of pyrophosphate as a universal approach to determine polymerase activity and assay polymerase inhibitors

**Journal:** Analytical Biochemistry

**Authors:** S. Malvezzi^‡^, S. J. Sturla^‡^, M. Tanasova^§*^

**Affiliations:**

^‡^Department of Health Sciences and Technology, ETH Zurich

^§*^Department of Chemistry, Michigan Technological University

**Contact email:** mtanasov@mtu.edu

**Abstract**

Fluorescence-based quantification of polymerase-mediated primer extension and single nucleotide incorporation based on the release of inorganic pyrophosphate (PPi) is described. The assay is efficient in quantifying polymerase kinetics in a laboratory or high-throughput settings and supports screening of compound libraries for identifying polymerase inhibitors. The assay has been applied to analysis of unnatural nucleotides as inhibitors of translesion synthesis (TLS) polymerases Dpo4 and Pol η during replication over DNA with drug-relevant adducts and shows for the first time the lesion-specific inhibition of TLS.

**Specifications Table**

| Subject area | *Chemical Biology* |
| --- | --- |
| More specific subject area | *Toxicology, Drug Development* |
| Type of data | *Table, text file, graph, figure* |
| How data was acquired | *Oligonucleotide synthesis, fluorescence assay, gel electrophoresis* |
| Data format | *Analyzed by Mass Spectrometry, Fluorescence plate-reader, and Molecular Imager Gel Doc XR+Imaging System from Bio-Rad* |
| Experimental factors | *Enzymes were contained in Tris buffer.* |
| Experimental features | *Fluorescent measurements were used to evaluate DNA synthesis. The data was correlated to that obtained with 1D gel electrophoresis.* |
| Data source location | *Zurich, Switzerland* |
| Data accessibility | *Data is provided in* Supplementary materials directly with this article. |

**Value of the data**:

- Quantitative fluorescence-based analysis of primer extension and single nucleotide incorporation efficiency.
- Quantitative fluorescence-based analysis translesion synthesis over DNA with drug-relevant adducts.
- Quantitative evaluation of DNA synthesis inhibition and identification of lesion-specific translesion synthesis inhibition.

1. **Data, Experimental Design, Materials and Methods**

We have established a fluorescence-based assay for quantitative monitoring of DNA synthesis based on the formation of pyrophosphate [1]. The assay was developed for the use with DNA containing different DNA alkylation products. DNAs were analyzed with Mass Spectrometry and the corresponding spectra are shown in Supplementary materials. Replication of modified and natural DNA templates by Y-family translesion synthesis polymerases was monitored and quantified based on fluorescence emission being proportional to pyrophosphate release. The data were correlated to those produced by monitoring replication of modified and natural DNA templates via gel electrophoresis, and the comparative data are shown in Supplementary materials. The data support the reliability of the assay and its applicability to high throughput screening of polymerase inhibitors. Validation of the assay with unnatural nucleosides resulted in the observation that lesion-specific inhibition of translesion synthesis is possible.

**1.1 Oligonucleotide characterization.**

The synthesized oligonucleotides were purified by HPLC and characterized by MS analysis (Electronic Supplementary Material, Fig.S1-4). 30mer-GG and 30mer-*O*^6^-MeG DNA templates were purified by HPLC (Agilent 1100 Series) with an Agilent Eclipse XDB-C18 5μm 4.6 x 150mm column. The chromatographic mobile phases were 50 mM triethylammonium acetate and acetonitrile. A gradient of acetonitrile from 8 to 12% over 30 min was used for 30mer-GG and from 9.5 to 12% over 49 min for 30mer-*O*^6^-MeG. For 30mer-Pt, a Phenomenex Luna C18 5μm 250 x 4.6mm column was used with an acetonitrile gradient from 5 to 12% over 75 min. The eluted fractions containing DNA templates were concentrated to dryness in a MiVac centrifugal evaporator (GeneVac), resuspended in deionized water and checked by direct injection into an Agilent MSD SL ion trap mass spectrometer with electrospray ionization. The template 30mer-3d-3MeA was purified by PAGE electrophoresis with a 20% (w/v) acrylamide/urea 7M gel followed by solid phase extraction with a Sep-Pak C18 Classic cartridge (Waters).

**
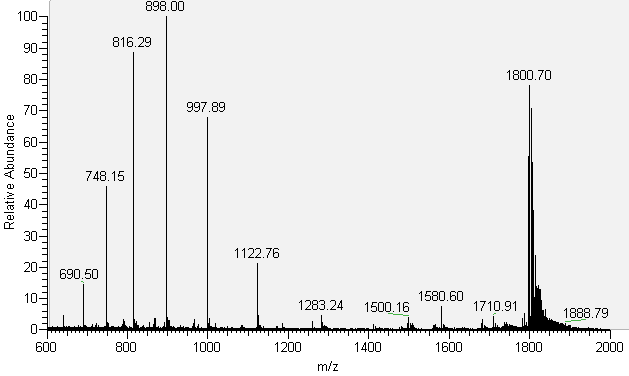
**

**Fig.S1** HPLC chromatogram and MS spectrum of 30mer-GG

**
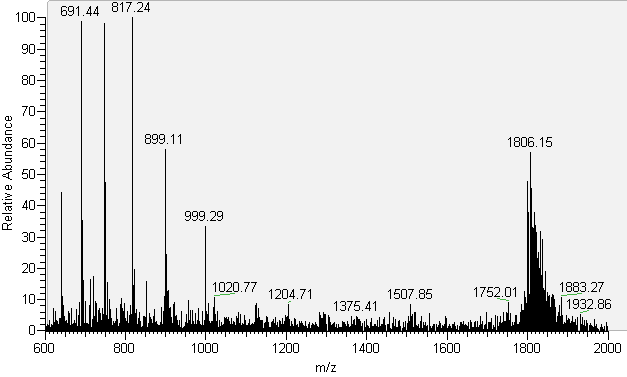
**

**Fig.S2** HPLC chromatogram and MS spectrum of 30mer-O^6^MeG

**
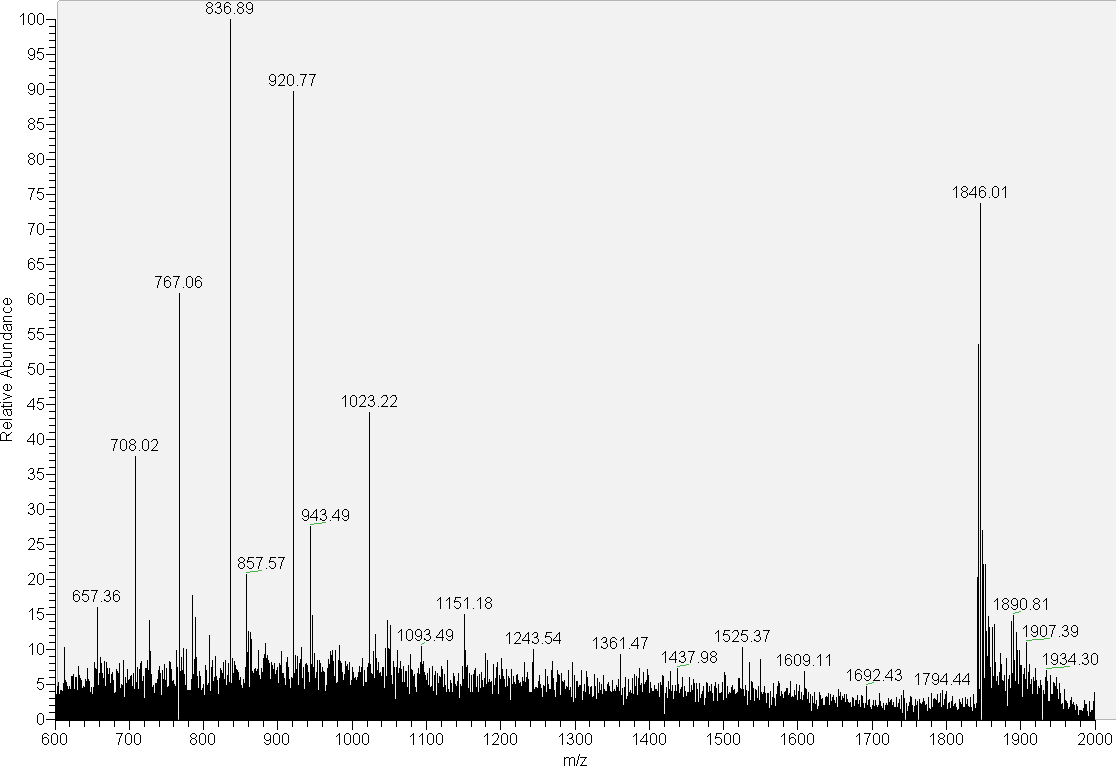
**

**Fig.S3** HPLC chromatogram and MS spectrum of 30mer-Pt


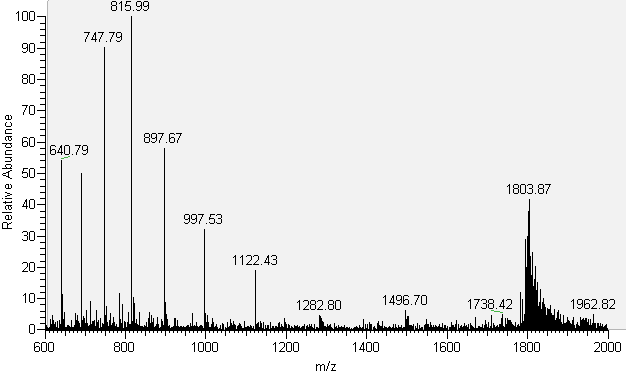


**Fig.S4** MS spectrum of 30mer-3d-3MeA

- 1. **Gel electrophoresis data**

Extent of primer extension for each reaction was monitored in parallel by denaturing gel electrophoresis for comparison with PE-PiPer data. 2 µl of the quenched primer extension reaction were combined with 3 µl loading buffer (95% formamide, 18 mM EDTA, 0.1% Bromophenol Blue) and electrophoresis was performed with a 20% (w/v) acrylamide/urea 7M gel with an XCell SureLock mini-cell electrophoresis system from Life Technologies at 300 V for 90 min. The gel was then stained in 1X SYBR Gold solution at room temperature for 20 min. Gel bands were quantified with a Molecular Imager Gel Doc XR+Imaging System from Bio-Rad.

**Fig.S5** Modulation of Pol η TLS activity by nucleotide analogs detected by gel electrophoresis. Reaction solutions contained 20 nM Pol η, 600 μM dNTPs and 60 μM nucleotide analogs. DNA templates (1.6 μM) were 30mer-GG (a), 30mer-O6MeG (b), 30mer-Pt (c) and 30mer-3d-3MeA (d). Graph bars represent the average of independent replicates and their standard deviation (n=3).

**References**

1. Malvezzi, S., S.J. Sturla, and M. Tanasova, *Data in Support of Quantification of Pyrophosphate as a Universal Approach to Determine Polymerase Activity and Assay Polymerase Inhibitors Analytical Biochemistry.* Anal. Biochem., 2015, *in press*.
